# Is it worth offering a routine laparoscopic cholecystectomy in developing countries? A Thailand case study

**DOI:** 10.1186/1478-7547-3-10

**Published:** 2005-10-31

**Authors:** Yot Teerawattananon, Miranda Mugford

**Affiliations:** 1 International health Policy Program, Bureau of Policy and Strategy, Ministry of Public Health, Nonthaburi, Thailand; 2 School of Medicine, Health Policy and Practice, University of East Anglia, Norwich, UK

## Abstract

**Objective:**

The study aims to investigate whether laparoscopic cholecystectomy (LC) is a cost-effective strategy for managing gallbladder-stone disease compared to the conventional open cholecystectomy(OC) in a Thai setting.

**Design and Setting:**

Using a societal perspective a cost-utility analysis was employed to measure programme cost and effectiveness of each management strategy. The costs borne by the hospital and patients were collected from Chiang Rai regional hospital while the clinical outcomes were summarised from a published systematic review of international and national literature. Incremental cost per Quality Adjusted Life Year (QALY) derived from a decision tree model.

**Results:**

The results reveal that at base-case scenario the incremental cost per QALY of moving from OC to LC is 134,000 Baht under government perspective and 89,000 Baht under a societal perspective. However, the probabilities that LC outweighed OC are not greater than 95% until the ceiling ratio reaches 190,000 and 270,000 Baht per QALY using societal and government perspective respectively.

**Conclusion:**

The economic evaluation results of management options for gallstone disease in Thailand differ from comparable previous studies conducted in developed countries which indicated that LC was a cost-saving strategy. Differences were due mainly to hospital costs of post operative inpatient care and value of lost working time. The LC option would be considered a cost-effective option for Thailand at a threshold of three times per capita gross domestic product recommended by the committee on the Millennium Development Goals.

## Introduction

It is widely accepted that laparoscopic cholecystectomy (LC) is the first-line treatment for uncomplicated gallstone disease in developed countries where up to 80% of all cholecystectomy is performed through laparoscopy [1-3].

In contrast to the conventional open cholecystectomy (OC), which is performed through an approximately 15-centimeter right sub-costal incision and commonly causes a serious degree of postoperative pain and longer hospital stay, LC is associated with a shorter hospitalization, more rapid return to work and better quality of life, at least in the short and intermediate term (3 years) after the operation[4].

The saving of hospital costs by LC is, however, not yet in agreement [5-8]] due to a higher operation cost which contributes around 60% of total hospital costs[9]. Furthermore, high complication rates were reported[10,11]. A meta-analysis also concludes that LC appeared to have a higher rate of common bile duct injuries[12].

In Thailand, since LC was first introduced in 1993, its adoption by healthcare providers has been relatively slow. By 2001, LC accounted for only 17% of the overall rate of cholecystectomy[13]. Some factors could explain the slow diffusion rate of the new procedure in Thailand. Firstly, the absence of financial incentives is believed to be a major cause. Under the capitation of the largest public insurance scheme, Universal Coverage Scheme (UC), OC has been reimbursed, but not LC. Thus, up to 10,000 Baht of co-payment by patients is needed if patients want laparoscopic surgery. Unsurprisingly, the proportion of UC patients undergoing LC, 13%, was the lowest across public health insurance schemes[13].

Patients under Civil Servant Medical Benefit Scheme (CSMBS) did not face the same financial disincentives. The CSMBS, covering government employees and their relatives, reimburses hospitals at a fixed rate for certain surgical procedures but, unfortunately, reimbursement rates set for OC and LC are alike, even though LC is associated with a shorter hospital stay. CSMBS reimburses the operation and hotel cost of hospital admission separately. A better socioeconomic status among CSMBS beneficiaries compared to UC may explain the greater LC uptake[14]. LC accounted for 28% of all cholecystectomy for these patients[13].

Secondly, the initial costs of setting up the equipment to perform LC and need for training for surgical team might have limited the ability of some hospitals to perform the procedure and these hospitals are likely to continue the status quo unless having strong support from Ministry of Public Health[13].

Given the lack of uptake and the greater potential cost-effectiveness, there is an urgent need to determine the value for money of LC compared to the traditional OC. Appropriate assessment of evidence may help to identify whether LC should be reimbursed under the Thai public insurance system. The aim of the present study is to investigate whether LC is a cost-effective strategy for managing gallbladder stone disease in the setting of Thailand. Although there are a number of economic evaluations on LC versus OC[6,7,15-18]], no single study was conducted in developing countries where cost burden may largely differ from those in western countries. The study is also conducted using societal perspective, which is recommended for public reimbursement[19], but rarely found in previous evaluations.

## Materials and methods

### Model

**Figure 1:**
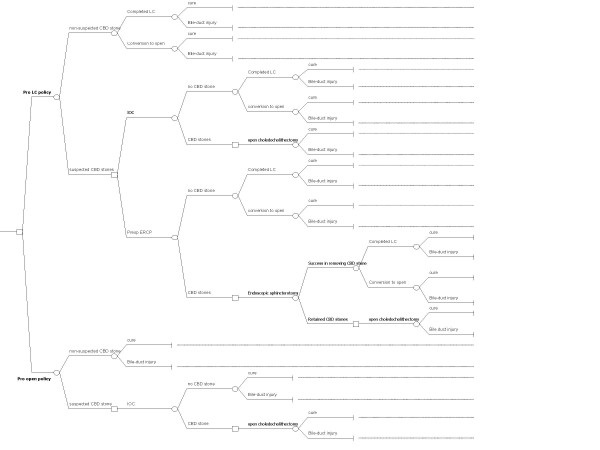
Decision tree illustrating the probable course of events for the management strategies being compared. A node in the represented by the box is the point of making decision between alternatives; nodes in the represented by the circle are points of events occurred.

A decision tree validated by a group of experts in Thailand was used to model all of the clinically important outcomes of two different strategies for treating of gallbladder stones--pro LC versus pro OC policy (Figure 1). The start of the decision tree is the case of a patient who is eligible for surgery for gallbladder stones--cholelithiasis, but the management strategy of cholelithiasis will differ based on whether or not common bile duct (CBD) stones are presented. Surgeons do not know with certainty, which of their patients with cholelithiasis actually have CBD stones. This is accounted for by explicitly modelling the effects of each strategy for the two different situations--one where physician suspects the existing of CBD stones e.g. acute pancreatitis, duct dilatation of CBD, obstructive jaundice[2,20,21] and one where there are no suspected signs and symptoms of having CBD stones.

The diagnosis of CBD stones or choledocholithiasis, could be done by ultrasonography, intravenous cholangiography, endoscopic retrograde cholangiography (ERCP) or Magnetic Resonance Image (MRI). Cholangiography and ERCP are most commonly used for definitive diagnosis in Thailand. Routine use of preoperative ERCP or intraoperative cholangiography (IOC) yields very little benefit for detection of CBD stones over and above that which is obtained with selective policies[16]. Hence, all patients without suspected CBD stones are directly assigned for LC or OC. Only those undergoing LC the result may be successful or unsuccessful (conversion from LC to OC). The final outcome is either cure or having bile-duct injuries.

For the patients with suspected CBD stones under pro LC policy, they will be assigned for ERCP or IOC depending on availability of the procedure. For those with positive IOC, open choledocholelithectomy is a choice of treatment. On the other hand, patients with positive ERCP undergo endoscopic sphincterotomy, and if it is unsuccessful, then open choledocholelithectomy is performed.

Under the pro OC policy the patients with suspected CBD stones receive IOC and if positive, open choledocholithectomy is the definitive treatment.

### Estimation of parameter probabilities

All parameters for the model were obtained from a systematic review of national and international literature, searching of Thai- and English-language studies. We identified all English language articles published up to year 2004 from Medline and EMBASE using searching keyword "cholecystectomy". The Thai literature, including related Masters and Docteral's dissertations, were also identified using electronic and manual approach from database at Library of Mahidol University. We initially reviewed all identified abstracts. The full articles were considered if their objectives are about to quantify mortality, morbidity, compliance and treatment costs.

The mortality outcome of the laparoscopic and open strategies was considered to be equivalent[12]. A higher rate of common bile duct injuries, but better quality of life in short and intermediate terms were demonstrated for LC[4,16]. A cost-utility analysis in terms of Baht per Quality Adjusted Life Year (QALY) was, therefore, selected as the analytical approach.

Table 1 presents the probabilities for all clinical outcomes in the model and the utility values, as well as the range tested in sensitivity analysis. For example, the probability of finding patients with suspected signs and symptoms of having common bile duct stones was set at 0.3440 and its standard error (SE) was 0.0123[22,23]. With the overall incidence of CBD stones with among patients with cholelithiasis at 10%, the probability of having common bile-duct stones among suspected cases was set at 0.293 with SE of 0.045.

**Table 1:** Corresponding transitional probabilities of epidemiological variables and the utility values

**Input Variables**	Point estimates (mean)	Standard error for uncertainty analysis	Data sources
*Epidemiological variables*			
Probability of having suspected signs of CBD stones	0.3439	0.0123	22–24
Probability of having CBD stones among suspected cases	0.2929	0.0455	1
Proportion of ERCP available for patients who need it	0.5000	0.0498	Expert opinion
Probability of conversion from LC to OC	0.0550	0.0010	12, 24, 26
Probability of bile duct injury among patients undergoing LC	0.0050	0.0003	12
Probability of bile duct injury among patients conversed from LC to OC	0.0030	0.0017	Expert opinion
Probability of bile duct injury among patients undergoing OC	0.0024	0.0004	12
Probability of bile duct injury among patients undergoing open explored CBD	0.0010	0.0010	Expert opinion
Probability of retained CBD stones after undergoing ERCP	0.1279	0.0358	24–26
*Utility variables*			
Utility of case with completed OC	0.80	0.02	16
Utility of case with completed LC	0.90	0.02	16
Utility of case with bile-duct injury in the first year	0.80	0.02	16
Utility of case with bile-duct injury in the subsequent twenty years	0.89	0.01	16

CBD = common bile duct

ERCP = endoscopic retrograde cholangiopancreatograpy

LC = laparoscopic cholecystectomy

OC = open cholecystectomy

Updating the meta-analysis conducted by Shea et al[12] the conversion rate from LC to OC was 0.055 (SE = 0.001) and the probability of bile duct injury among patients undergoing LC and OC were 0.0050 (SE = 0.0003) and 0.0024 (SE = 0.0024) respectively. A consensus from the Thai expert panel meeting to review the model indicated that a probability of bile duct injury among patients conversed from LC to OC should be something in between the probabilities of injury among patients undergoing OC and the probabilities of injury among patients undergoing LC. We assumed the probability of 0.0030 and SE of 0.0017. The panel also indicated that a probability of bile duct injury among patients undergoing open explored CBD should be the lowest. We set the rate and its standard error of 0.001. The meta-analysis of studies by Sangsubhan et al[24], Rhodes et al[25], and Konstadoulakis et al[26] found a probability of retained CBD stones after undergoing ERCP at 0.128 (SE = 0.0358).

### Utility

Laparoscopic cholecystectomy was superior to open cholecystectomy in enhancing the quality of life for all eligible patients. The most comprehensive quality of life assessment study found from our review is of Cook et al[16]. They conducted a prospective assessment from 96 members of the general public in Melbourne using a standard questionnaire of which its questions included frequency and intensity of clinical factors e.g. pain, nausea, vomiting, identified from previous patient's interviews. We used their utility values and confidential intervals adjusted by 12-month values as our utility inputs (In fact, Topcu et al[4] indicated the difference in utility between LC and OC at 36 months. We took conservative estimation, assuming the difference of utility lasted by 12 months.)

### Resource use parameters

In this study both direct and indirect costs borne by health care providers and households were collected in two ways: patient questionnaire interviews and data extraction from hospital case notes.

Chiang Rai regional hospital was purposively selected for costing study. This was the only public hospital that provided free of charge of LC and OC for UC beneficiaries, while other public hospitals offered only OC free. The prospective reviews of medical records of all patients undergoing open or laparoscopic cholecystectomy during September to November 2004 were performed. We also conducted a retrospective medical record review for rare conditions i.e. open choledocholithectomy, cases of conversion from LC to OC, treatments for CBD injuries, and endoscopic sphincterotomy, which had occurred in the past two years (October 2002-September 2004).

The case note review aimed to measure hospital resources consumed (e.g. clinician's time; type and number of investigations, drugs, and disposable equipments; length of hospital admission) in excess of usual preoperative, operative, and postoperative care. To ensure comparability of the groups, exclusion criteria were defined in such a way that an OC could be compared with a LC. Of a total of 80 medical records reviewed, 48 records (60%) of acute cholecystitis patients initially presented with peritonitis, sepsis, neoplasm, or co-existing conditions i.e. uncontrolled diabetes mellitus, uncontrolled hypertension were excluded from the analysis.

The valuation of the hospital costs, including capital and overhead costs, for each individual patient was determined from each department involved in the routine services for patients with gallstones disease.

We completed micro-cost analysis of 15 cases of OC, 17 cases of LC, 7 cases of open choledocholithectomy, 3 case of conversion from LC to OC, 3 case of treatment of CBD injuries, and 1 case of endoscopic sphincterotomy. The mean age was 60.8 years for patients underwent OC and 58.1 years for LC, there was no significant difference (using the student t-test, p > =0.05).

Thirty-two patients who had the operation in October and November 2004 were also contacted to determine indirect costs. Daily event and cost questionnaires were used to collect patient-specific information for estimation of other household expenses e.g. travel costs, food, accommodation and opportunity loss from providing informal care and visits of relatives and friends at hospital and home, patient's recovery time to full activity after surgery. We conducted face-to-face interviews with patients at the post-operation visit (2–4 weeks after operation). For the patients who did not get back to the hospital at all or having longer follow-up period than 4 weeks, we used telephone interview to collect that data.

Because of very low complication rates and no available information on indirect costs related to complications, we assumed in the model a similar indirect cost for patients with and without complications.

The costs were represented in the model in Thai Baht in 2004 (40 Baht = 1USD or 75 Baht = 1 GBP). All costs and outcomes occurred beyond one year were discounted using the same rate of 3.5%.

### Uncertainty analysis

To determine if values within a plausible range for all input variables resulted in a different conclusion, we undertook probabilistic uncertainty analysis, assigning a beta distribution for all probability and utility parameters and gamma distribution for all cost parameters, and generating 1,000 rounds of simulations using Microsoft Excel^® ^with macro function on all estimated quantities.

The cost-effectiveness acceptability curve based on the net benefit approach is also provided to present the relation between the values of the ceiling ratio (willingness to pay for a unit more of QALY) and probability of favouring each treatment strategy.

## Results

### Costs

Table 2 summarises all important cost parameters for use in the economic evaluation model. It is worth noting that the average hospital cost for OC in all 15 patients (9,355 Baht per OC case, SE = 717) was lower than the average cost for LC in all 17 cases (20,790 Baht per LC case, SE = 507). However, length of hospital stay and time to full recovery were markedly reduced in patients undergoing LC (mean 3.8 days, SE 0.4) compared to those having OC for LC (mean 6.1 days, SE 0.5) and, therefore, made a much lower indirect cost (mean 8,617 Baht for LC versus 14,484 Baht for OC).

**Table 2:** Costs (per patient) related to open and laparoscopic cholecystectomy, in 2004 Thai Baht, and used as inputs in the model

	Open cholecystectomy		Laparoscopic cholecystectomy	
Variables	Mean	Standard error	Mean	Standard error
*Direct costs*				
Cholecystectomy (pre-, intra-, and post-operation)	9,355	717	20,790	507
Conversion from Laparoscopic to open cholecystectomy			25,782	1,518
Intraoperative cholangiography (IOC)	1,502	154	1,502	154
Endoscopic retrograde cholangiopancreatography (ERCP)			2,011	120
Open choledocholithectomy	15,201	1,590	15,201	1,590
Endoscopic sphincterotomy			9,923	639
Treatment bile duct injury	12,068	2,926	12,068	2,926
Self-prescriptions and visiting private clinics (after discharge from hospital)	566	204	567	249
*Indirect costs*				
Foods, accommodations, and lost working and spare times, on the part of relatives during admission	7,519	383	3,810	759
Lost working and spare times, on the part of relatives after discharging from hospital	2,945	1,409	2,693	650
Lost working and spare times, on the part of patients (on a whole course)	4,008	1,004	2,069	488
Transfer costs (i.e. sick compensations)	69	55	17	12

### Cost-utility

Programme costs and outcome at base-case scenario for each treatment strategy are demonstrated in table 3. The programme costs are lower for pro OC policy in both using government's and societal viewpoints. However, the gap of programme costs (incremental cost) between the two strategies is 42% [(12,000-7,000)/12,000] smaller in the use of a societal viewpoint.

**Table 3:** Deterministic results from the model

	Open cholecystectomy	Laparoscopic cholecystectomy	Incremental values
Programme cost using government perspective	11,000	23,000	12,000
Programme cost using societal perspective	26,000	33,000	7,000
Programme effectiveness (QALYs)	0.798	0.885	0.087
Cost per QALY using government perspective			134,000
Cost per QALY using societal perspective			89,000

Note: the costs and incremental values were given to nearest 1,000 Baht price level

Pro OC and LC strategy provide 0.798 and 0.885 QALYs, respectively. On the other hand, moving from a cheaper and lower effectiveness strategy, pro OC, to pro LC policy yields extra 0.087 QALYs.

When only direct costs were compared, an incremental cost per QALY of moving from OC to LC is 134,000 Baht. The incremental cost-effectiveness ratio decreases to 89,000 Baht per QALY when including indirect cost of a wider societal perspective. Moving from OC to LC would add a financial burden of 12,000 Baht per case to the government but offset the indirect costs of 5,000 Baht that is presently shouldered by the households.

### Uncertainty analysis

**Figure 2:**
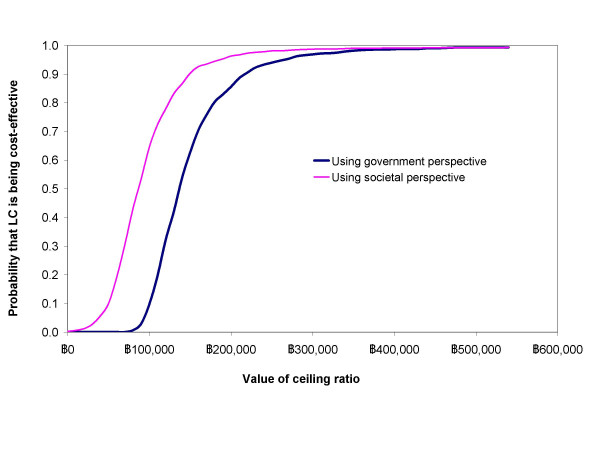
Cost-effectiveness acceptability curves using net benefit approach.

The cost-effectiveness acceptability curves in figure 2 summarise the robustness of the model regarding uncertainty estimation of the programme cost and effect for each treatment strategy. At the zero ceiling ratio, indicating that no further resources would be allocated to healthcare, pro OC policy is a dominant strategy, particularly using a government perspective. When the ceiling ratios are greater than 90,000 and 140,000 using government and societal perspective respectively, pro LC policy becomes a preferable choice, offering a better chance of saving one QALY at given money. However, the probabilities that LC outweighed OC are not greater than 95% until the ceiling ratio reaches 190,000 and 270,000 Baht per QALY using societal and government perspective respectively.

## Discussion

The study provides the same results using the two different viewpoints of the analysis; LC is a more expensive but likely to provide a better quality of life than OC. At base-case scenario the ratio of extra cost of LC to its extra utility gained varies between 89,000 and 134,000 Baht per QALY, depending on whether indirect costs are included.

To make the model as simple as possible and to focus only on a comparison between the use of OC and LC, the model has a limitation about retained CBD stones between the two strategies. Retained CBD stones could arise from a difference in the use of ERCP and IOC between the two approaches. However, estimating the sensitivity and specificity of IOC and ERCP is not straightforward since the two investigations and surgery are not performed simultaneously, so it is possible that the stones can migrate out of the CBD spontaneously in the interval between investigation and surgery, or that additional stones enter the CBD from the gallbladder. The review of literature by Urbach et al[27] indicates that both IOC and ERCP provided the same specificity in detecting CBD stones but ERPC had superior sensitivity to IOC (95% vs 89%). In the other words, IOC would provide additional 5 false negative cases compared to ERCP. The literature also reveals that not all retained stones would be a problem but only 15% of these would go on to cause clinical problems[21]. Measuring QALYs for the retained CBD stones is also problematic. We found no study assessing utility of retained CBD stones.

The results of the economic evaluation for management options for gallstone disease in Thailand are irrelevant to comparable previous studies conducted in developed countries of which Cook J et al[16] and Berggren et al[7] found that LC was a cost saving strategy in comparison to OC. The difference could be explained with two reasons. Firstly, a higher wage rate for both health professionals and patients in US and Australia caused a significant higher hospital admission costs and opportunity cost of taking sick-leave. Secondly, the indirect costs quantified by the two studies are an overestimation, especially when applied to the Thai context. Since, following guidance on estimating 'friction costs' of lost work[28], the studies estimated the costs by multiplying average employment costs with proportion of population in the work force, but our study found that only 50% and 53% of patients undergoing open and laparoscopic cholecystectomy were active workforce members. However, it may be important to add a comment that this approach does not value the time of people not in paid work, even though many are productive members of society, e.g. self employed farmers, or unpaid house works.

Similar to the previous study[9], an operation cost contributed to 75% of total hospital costs for LC compared to 20% for OC. More than two third (70%) of operation cost was from operative instruments, although the sample hospital was quite efficient in using these instruments since, where possible, reusable instruments were introduced. Thus, we believe there is a little room to make the operation cost smaller.

To allow for the extra quality of life gained from LC, however, additional funding would be required. The judgement about whether to advocate LC over OC in such situations would depend on what benefit could be obtained from the use of these extra resources elsewhere. However, a broad comparison across health care interventions is unlikely due to the fact that it would have had an enormous work of analysis of possible treatments.

Another approach is setting a ceiling value for health benefit that society is willing to pay. The committee for development of Millennium Development Goals recommends the use of three times of Gross Domestic Product (GDP) per capital as a threshold for the consideration in developing countries. This application would presently lead to a ceiling value in Thailand of 270,000 Baht. In this case, LC is a cost-effective intervention. If extra funds could not be obtained for the health system directly, resources would have been obtained from elsewhere e.g. a co-payment system.

Furthermore, economic evaluations are commonly criticised by decision makers for ignoring budget impacts, about which decision makers desperately concerned. Payers can get into financial difficulty if they adopt too many cost-effectiveness interventions[29] and affordability, which depends on the overall volume of patients, is therefore a prime concern. We projected the financial implication if pro LC policy is adopted in Thailand (table 4). Assuming that 80% of patients who need cholecystectomy eligible for LC, the government would require 96 million Baht for supporting pro LC policy. At the same time, households could save 40 million Baht from indirect medical care cost resulted in the net of 56 million Baht required by society as a whole.

**Table 4:** Estimated financial burden on government budget and off-set cost by households if pro LC policy was adopted

Type of Insurance	Estimated cases in 2005 (a)	Estimated cases in 2005 (b)	Incremental financial burden of moving from OC to LC by government (c)	Household's off-set cost from moving from OC to LC (d)	Net financial burden to society (e)
UC	8,000	6,400	76,800,000	32,000,000	44,800,000
CSMBS	2,000	1,600	19,200,000	8,000,000	11,200,000

Total	10,000	8,000	96,000,000	40,000,000	56,000,000

UC = Universal Health Insurance Scheme

CSMBS = Civil Servant Medical Benefit Scheme

Calculation:

(b) = (a) × 0.8

(c) = (b) × 12,000

(d) = (b) × (12,000-7,000)

(e) = (c) - (d)
